# Efficacy of repetitive transcranial magnetic stimulation on postoperative pain in patients undergoing video-assisted thoracoscopic surgery: study protocol for a prospective, single-center, randomized controlled trial

**DOI:** 10.3389/fmed.2026.1756881

**Published:** 2026-03-19

**Authors:** Chunxiao Wang, Huadong Ni, Jiayu Yue

**Affiliations:** First Hospital of Jiaxing, Jiaxing, China

**Keywords:** acute postoperative pain, neuromodulation, perioperative analgesia, repetitive transcranial magnetic stimulation, video-assisted thoracoscopic surgery

## Abstract

**Background:**

Video-assisted thoracoscopic surgery (VATS) is a key treatment for many cardiothoracic issues, but intraoperative factors can lead to acute postoperative pain (APSP), impacting recovery and quality of life. While multimodal analgesic methods are used, they have notable side effects. Repetitive transcranial magnetic stimulation (rTMS) offers a non-invasive pain relief option by stimulating the primary motor cortex (M1), effective in chronic pain management. However, its role in preventing and treating APSP after VATS needs confirmation through high-quality clinical trials.

**Methods/design:**

In this single-center, prospective, double-blind, randomized controlled trial, a total of 260 patients slated for VATS will be randomly allocated in a 1:1 ratio to either an active rTMS group or a sham rTMS group. Both groups will receive a 20-min intervention at two specified time points: (1) the afternoon before the surgery while in the ward, and (2) 30 min before the surgical procedure on the day of the operation. The primary outcome measure is the incidence of moderate-to-severe pain at 24 h following surgery. Secondary outcome measures encompass pain scores at rest and during movement at specified time points (T1: 2 h, T2: 6 h, T3: 12 h, T4: 24 h, T5: 48 h, T6: 90 days postoperatively), total consumption of rescue analgesics, quantified in morphine milligram equivalents (MME) within the initial 48 h, time to first request for patient-controlled intravenous analgesia (PCIA), extubation duration, length of stay in the post-anesthesia care unit (PACU), and the Quality of Recovery-15 (QoR-15) score at 24 and 48 h postoperatively. All outcomes will be assessed utilizing validated instruments.

**Discussion:**

APSP remains a significant clinical issue, affecting patient outcomes and quality of life. rTMS, a promising non-invasive technique, has shown potential in treating mental disorders, neurological conditions, and chronic pain. This study aims to provide strong evidence on the effectiveness of rTMS in reducing acute pain after VATS, potentially enhancing multimodal postoperative pain management strategies.

**Clinical trial registration:**

[http://www.chictr.org.cn], identifier [ChiCTR2500111517].

## 1 Introduction

The extensive adoption of video-assisted thoracoscopic surgery (VATS) marks a significant advancement in the field of thoracic surgery. Compared to traditional thoracotomy, VATS offers numerous advantages, such as reduced postoperative pain, faster patient mobilization, shorter hospital stays, better preservation of lung function, and oncological outcomes comparable to those of tumor resection. These advantages collectively contribute to a substantial reduction in perioperative trauma, attributable to the minimally invasive nature of the procedure (1–3). Nonetheless, the term “minimally invasive” should not be misconstrued as synonymous with “pain-free.” A significant body of clinical evidence demonstrates that between 50% and 75% of patients undergoing VATS experience moderate to severe acute postoperative pain (APSP), with pain intensity potentially comparable to that associated with thoracotomy (4, 5). This pain arises from multiple mechanisms: direct compression and injury to the intercostal nerves by trocar ports, persistent stimulation of the parietal pleura and intercostal nerves by chest tubes, potential muscle spasms from surgical manipulation, and the unavoidable release of inflammatory mediators (6, 7). Inadequate control of this complex and intense acute pain has consequences extending far beyond subjective patient discomfort. This is a key factor leading to reduced lung capacity, diminished coughing ability, and retained secretions, which directly increases the risk of atelectasis and pneumonia (8, 9). Furthermore, pain restricts early ambulation, delays overall recovery, prolongs hospitalization, and increases healthcare costs. More profoundly, severe acute pain is a potent catalyst for central sensitization and is the strongest predictor for the development of chronic postsurgical pain, causing long-term physical and psychological distress (10). Therefore, optimizing APSP management after VATS is not only a humanitarian imperative but also crucial for improving outcomes, conserving healthcare resources, and preventing the transition to chronic pain.

Current standard practice for post-VATS analgesia involves multimodal strategies, typically combining regional anesthetic techniques such as paravertebral block or thoracic epidural analgesia with systemic analgesics including opioids, acetaminophen, and non-steroidal anti-inflammatory drugs (11–13). Although this approach has achieved some success, its limitations are increasingly apparent. Regional techniques carry risks of failure, operator dependency, limited duration of action, and potential complications like hypotension, nerve injury, and pneumothorax (14). Systemic opioids, as the cornerstone of analgesic therapy, pose significant clinical challenges due to their side effects: respiratory depression directly threatens pulmonary function in thoracic surgery patients; nausea, vomiting, intestinal obstruction, and constipation severely compromise rehabilitation quality and prolong hospital stays; long-term use carries addiction risks, thereby exacerbating global public health crises (15, 16). Clearly, relying solely on existing regimens makes perfect analgesia challenging to achieve. There is a pressing clinical need for innovative adjunctive therapies that work via different mechanisms, directly modulating central nervous system pain processing to reduce reliance on opioids.

Repetitive transcranial magnetic stimulation (rTMS), a non-invasive neuromodulation technique, generates controlled electrical currents in specific cortical regions, thereby enabling the permanent modulation of a group of neurons’ excitability (17, 18). rTMS has established therapeutic efficacy for conditions such as stroke sequelae, Alzheimer’s disease, Parkinson’s disease, and sleep disorders (19–21). In pain medicine, rTMS has been shown to target and modulate the “pain matrix,” including the primary motor cortex (M1), prefrontal cortex, anterior cingulate cortex, and thalamus (22). High-frequency (≥5 Hz) stimulation of the M1 region has been demonstrated to effectively treat chronic neuropathic pain and fibromyalgia (23). Its analgesic mechanisms are likely multi-faceted: (1) direct activation of M1-thalamic projections, modulating thalamic gating; (2) Enhancement of descending pain -suppressive pathways engaged by the gray matter surrounding the ventricles and the anterior ventral midline of the medulla; (3) facilitation of endogenous opioid system release; and (4) Induce synaptic plasticity changes (such as long-term potentiation or inhibition) to remodel abnormal pain networks (24–26). Given the established efficacy of rTMS in chronic pain, shifting its application earlier to the perioperative period to prevent or mitigate central sensitization and acute pain triggered by the potent noxious stimulus of surgery represents a potential paradigm shift and a logical scientific extension. Through pre-emptive or protective analgesia administered preoperatively or early postoperatively, rTMS might raise pain thresholds, rendering the central nervous system less responsive to noxious inputs.

Despite this strong rationale, the application of rTMS for controlling acute nociceptive pain following surgery remains a frontier area with scarce evidence. Existing research predominantly focuses on chronic pain, with high-quality studies targeting acute pain, especially after high-pain-intensity surgeries like thoracic procedures, being exceptionally rare.

On the basis of the above content, we formulate the core hypothesis of this study: A series of 10-Hz rTMS treatments administered to areas M1 during the perioperative period for VATS can safely and effectively reduce central pain sensitization, thereby significantly attenuating APSP intensity, decreasing rescue opioid consumption, and ultimately enhancing overall recovery quality.

## 2 Methods and analysis

### 2.1 Study design

**FIGURE 1:**
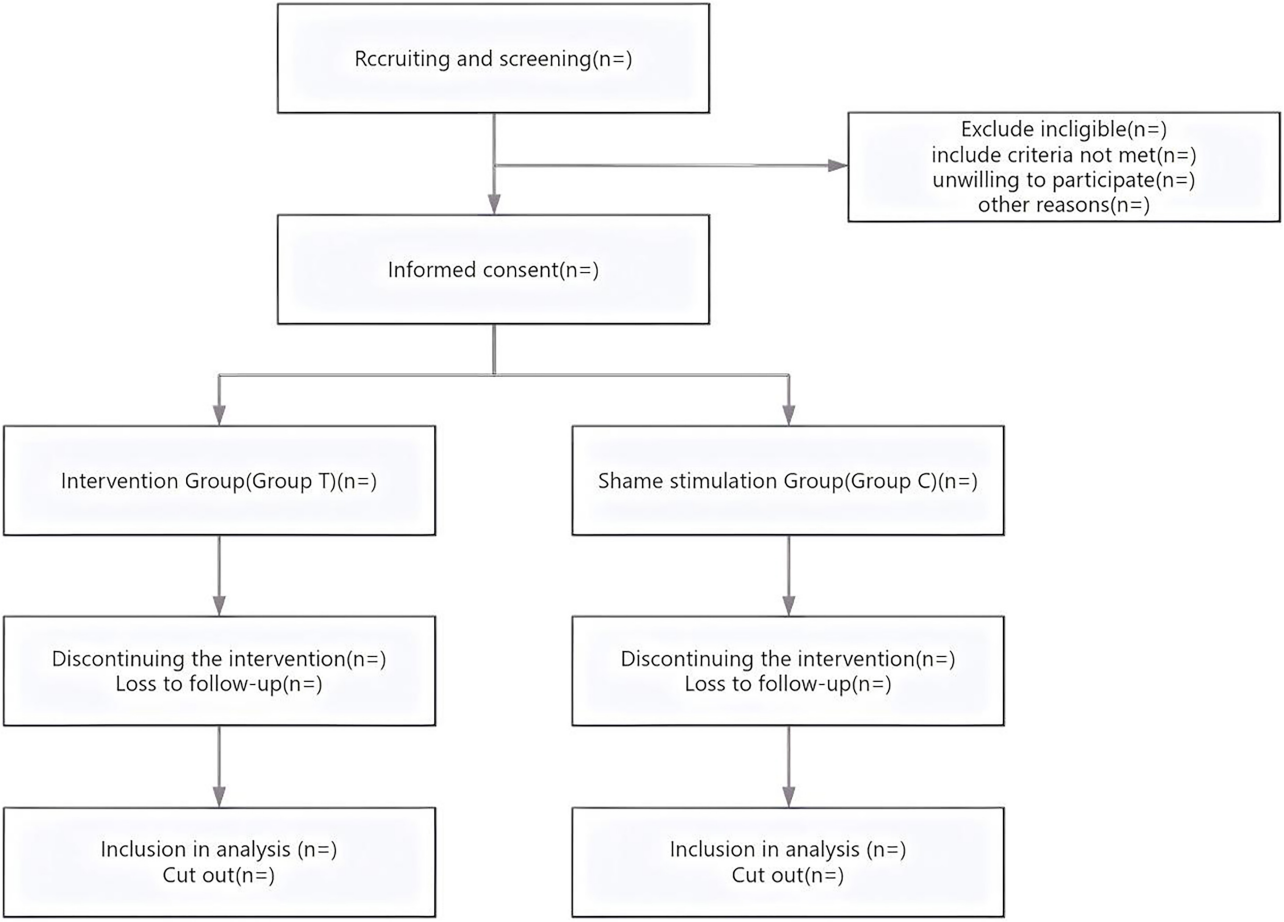
Flow diagram of the study.

A single-center, prospective, randomized controlled clinical trial conducted at the Affiliated Hospital of Jiaxing University. The study plans to enroll 260 patients scheduled for elective thoracoscopic surgery. This research protocol has been granted approval by the Ethics Review Committee of Jiaxing University Affiliated Hospital (Approval No. 2025-KY-630) and has been registered with the China Clinical Trial Registry (http://www.chictr.org.cn, Registration number: ChiCTR2500111517). Recruitment of participants is currently underway, strictly adhering to predetermined inclusion and exclusion criteria. All participants will sign written informed consent forms prior to enrollment. This study complies with the Standard Protocol Items: Recommendations for Interventional Trials (SPIRIT) guidelines (27). The flow of patient recruitment, intervention, and outcome measurement is summarized in Figure 1.

### 2.2 Eligibility criteria

Patients at Jiaxing University Affiliated Hospital who meet the criteria for VATS will undergo screening and evaluation by the research team based on predefined standards the day before surgery. Written informed consent will be provided prior to the baseline assessment (Supplementary Material 1).

### 2.3 Inclusion criteria

(1). Aged ≥ 18 years, gender not limited;
(2). ASA classification I–III;
(3). Proposed elective unilateral thoracoscopic surgery;
(4). Voluntarily participate in this study and sign an informed consent form.

### 2.4 Exclusion criteria

(1). Contraindications for rTMS (such as intracranial metal implants, pacemakers, personal or family history of epilepsy);
(2). Long-term use of opioids, sedative medications, or a diagnosis of chronic pain syndrome;
(3). Severe mental or cognitive dysfunction that prevents them from communicating or cooperating with the assessment;
(4). History of drug or alcohol abuse;
(5). Pre-operative pre-existing neurological deficits;
(6). Pregnant or breastfeeding women;
(7). Any other circumstances that the researcher considers unsuitable for participation in this study.

### 2.5 Discontinuation criteria

(1). Intraoperative conversion to thoracotomy or extended surgical scope (e.g., pneumonectomy, pleuropneumonectomy).
(2). Postoperative ICU stay exceeding 24 h, preventing effective assessment.
(3). Patient or family requests withdrawal at any stage.
(4). A serious adverse event occurred, leading the investigator to determine that the intervention must be terminated.

The participants have the privilege to drop out of the study at any time for any reason, and this will not affect their routine medical care. The research team will ensure appropriate care continues to be provided and will document in detail the reasons for withdrawing from the study. Additionally, the investigator may discontinue a participant’s involvement in case of severe organ dysfunction, protocol non-compliance, or occurrence of serious adverse events (SAEs).

### 2.6 Randomization and blinding

Using a random block allocation method (block sizes of 4 and 6), subjects were assigned in a 1:1 ratio to either the active or sham rTMS group. The sequence for randomization will be produced by an individual statistician who is not involved in the recruitment of patients or the assessment of outcomes, using SAS software (version 9.4; SAS Institute, Cary, North Carolina, United States). This serial number will be enclosed in a sequentially numbered, impervious to light, sealed envelope. Upon eligibility confirmation and consent, the researcher will open the next envelope in sequence and execute the assignment. Sequentially numbered, opaque, sealed envelopes will be opened only after participant enrollment and consent. The envelope will not be resealed. To assure compliance with the Blind Method, patients will be informed during preoperative consultations about potential sensations experienced during therapy, including tapping, pricking, warmth, or vibration. Patients will also be explicitly advised that the intensity of these sensations varies among individuals and should not be used as a basis for evaluating treatment efficacy. The stimulation devices are identical in appearance; the only difference lies in the placement angle of the coil during stimulation. The device is to be managed by independent researchers not participating in the study, and the screen is to remain off prior to treatment. Each participant will complete a questionnaire after the intervention to infer group assignment, with data reported and analyzed using the Bang blind index. All personnel involved in outcome assessment, data analysis, surgery, and the participants themselves will be blinded to group assignment. The outcome evaluators will undergo rigorous blinding training prior to the study commencement to familiarize themselves with operational processes and standards, thereby minimizing the impact of subjectivity on results.

### 2.7 Unblinding

During the intervention phase, participants will undergo systematic evaluation to screen for potential rTMS-related complications or life-endangering symptoms. Prospective undesirable events include seizures, syncope, vagal reactions, headache, and auditory discomfort. The principal investigator may unblind the study only when complications requiring urgent medical intervention arise.

### 2.8 Anesthesia management

Patients receive standard preoperative educational counseling and anesthesia consultation, including medical history review, explanation of the anesthesia plan, and disclosure of potential risks. They sign informed consent forms for anesthesia and the experimental procedure. Once in the operating room, peripheral venous access is established, and noninvasive blood pressure, electrocardiogram, and pulse oximetry are monitored. After a negative Allen’s test, radial artery cannulation will be performed under local anesthesia with lidocaine for arterial blood gas analysis and invasive blood pressure monitoring. Bispectral index (BIS) electrodes will be placed on the forehead for depth of anesthesia monitoring.

Anesthesia induction will be achieved via intravenous administration of propofol (1.5–2 mg/kg), sufentanil (0.1–0.5 μg/kg), and cisatracurium (0.15 mg/kg). A properly sized double-lumen endotracheal tube or bronchial blocker will be inserted via rapid sequence intubation. Initial mechanical ventilation parameters will be set as follows: tidal volume 6–8 mL/kg, respiratory rate 12 breaths/minute, FiO_2_ 1.0, maintaining end-tidal carbon dioxide pressure (PETCO_2_) within the range of 35–45 mmHg. Maintenance anesthesia employed a combined intravenous-inhalation technique: continuous propofol infusion at 4–6 mg/(kg⋅h), continuous remifentanil infusion at 8–10 μg/(kg⋅h), supplemented with 1% sevoflurane inhalation (targeting a brain-stem evoked potential BIS of 40–60). Vasopressors will be administered to maintain mean arterial pressure and heart rate within 20% of baseline values. Cisatracurium (0.05 mg/kg) will be given as needed on an intermittent basis.

Discontinue sevoflurane inhalation 30 min prior to the end of surgery.

All anesthetic agents will be discontinued upon completion of the procedure. The patient will be moved to the post-anesthesia care unit (PACU). Postoperative patient-controlled intravenous analgesia (PCIA) will be administered: Sufentanil 2 μg/kg + Ondansetron 0.2 mg/kg + Normal Saline to a total volume of 100 ml; baseline infusion rate 2 ml/h, PCA bolus dose 0.5 ml, with a 15-min lockout interval. Extubation criteria: when the patient can respond to verbal commands, regain muscle strength, and demonstrate satisfactory respiratory parameters. Patients with a Steward score > 4 can be transferred to the ward. Rescue analgesia (intravenous tramadol 50 mg) will be administered if the NRS pain score exceeds 4.

### 2.9 Study intervention

**FIGURE 2:**
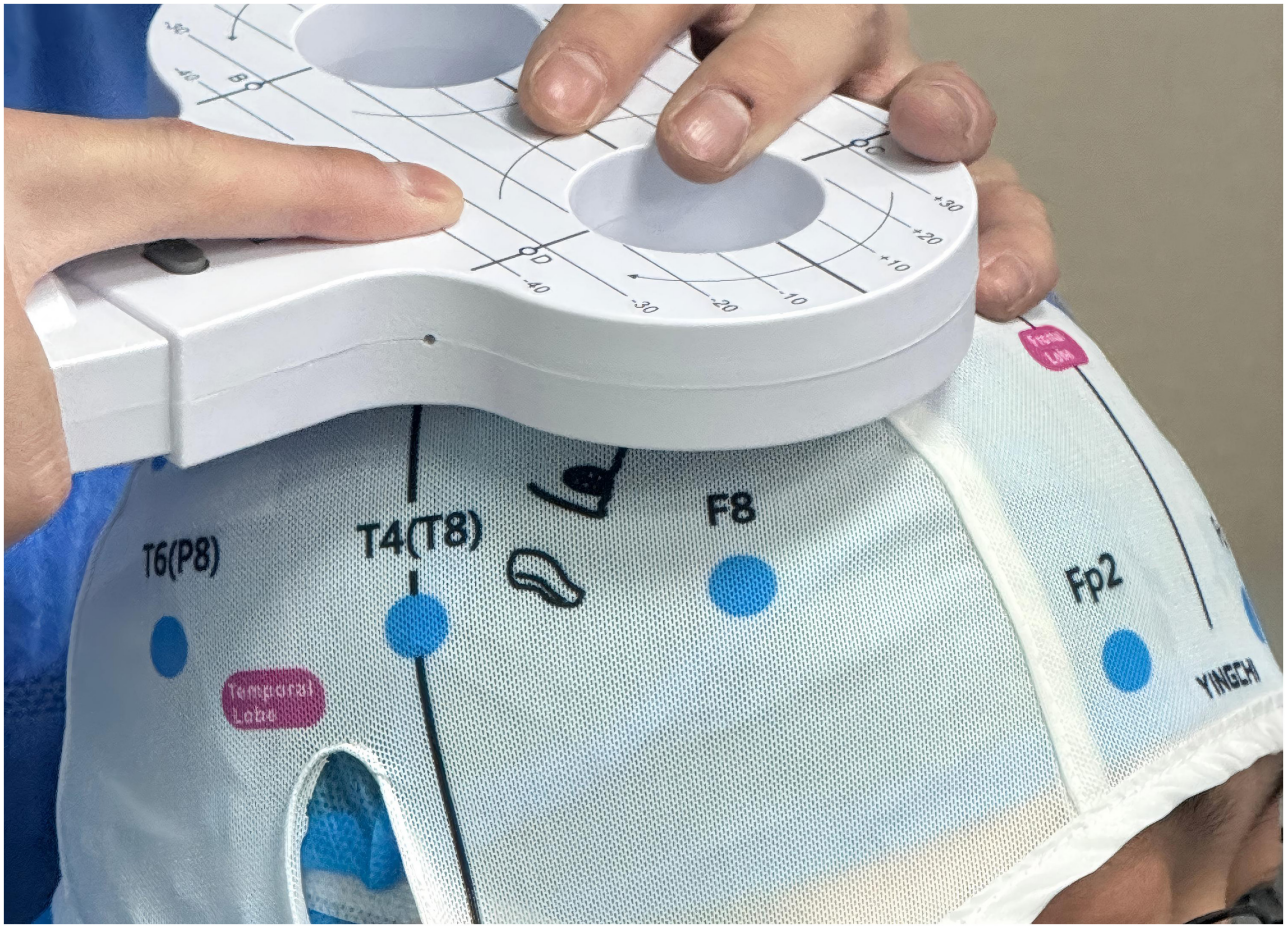
The magnetic stimulation coil was positioned over the M1 location. Transcranial magnetic stimulation.

During the morning of the day before surgery, researchers will identify prospective participants based on the surgical schedule. Interventions will occur at two time points (Table 1): (1) First intervention: Afternoon before surgery in the ward. (2) Second intervention: 30 min before surgery on the operation day. The Mag TD rTMS device (Yiruide Medical Equipment, Wuhan, China) with its figure-of-eight coil will be used. We chose the left M1 region as the stimulation target because the left hemisphere is the dominant hemisphere for most people, and its neuroplasticity and regulatory potential may be stronger. A study published in 2024 confirmed that rTMS targeting the left M1 can selectively increase the concentration of β-endorphin in plasma. β-endorphin is one of the strongest “natural painkillers” in the human body. This provides direct neurochemical evidence for left M1 stimulation (24). Therefore, in research, to unify standards and reduce variables, the left M1 has become the most commonly used target with the most comprehensive research data (28). For the group receiving effective rTMS, stimulation of the M1 region will be performed by an independent investigator not involved in this study (as shown in Figure 2). M1 localization will utilize the international 10–20 electroencephalography system (29). This location is situated approximately 5 cm posterior-lateral and 6 cm anterior to the vertex of the head, and can be confirmed using a positioning cap. Single-pulse transcranial magnetic stimulation is employed for precise localization to determine the optimal stimulation point capable of eliciting the most stable motor-evoked potential (MEP) in the contralateral flexor digitorum superficialis muscle. As clinical studies on rTMS for APSP are limited, stimulation parameters are based on preclinical evidence. The stimulation frequency will be 10 Hz, intensity at 90% of the resting motor threshold (RMT). Each train will last 10 s, followed by a 20-s inter-train interval, for 30 trains totaling 3,000 pulses. RMT is a defined by the minimum stimulus intensity (expressed as a percentage of maximum output) required to elicit a MEP of >50 μV in at least five out of 10 consecutive trials while the target muscle is in a relaxed state (30). For the sham rTMS group, the device and coil placement will be identical, but the coil will be tilted 90° away from the scalp. This produces similar auditory and somatosensory cues (sound, vibration) without delivering effective magnetic stimulation to the cortex, serving as a placebo control (31). Professionally trained researchers will strictly follow operating procedures and closely monitor participants’ responses throughout the intervention to make sure no adverse reactions occur. Should participants report intolerable discomfort, the stimulus will be immediately discontinued. A limitation of this approach is that pseudo-stimulation achieved through 90° coil tilt may not fully match scalp sensations, and depending on the geometry, it may still induce partial cortical stimulation.

**TABLE 1:** The study schedule for enrollment, treatments, outcome measurements, and data collection.

Study period									
Time point	Enrollment		Post-randomization						
	Pre-2	Pre-1	30 min before surgery	2 h after surgery (T1)	6 h after surgery (T2)	12 h after surgery (T3)	24 h after surgery (T4)	48 h after surgery (T5)	90 days after surgery (T6)
Enrollment									
Inclusion criteria	✓	–	–	–	–	–	–	–	–
Exclusion criteria	✓	–	–	–	–	–	–	–	–
Informed consent	✓	–	–	–	–	–	–	–	–
Randomization	–	✓	–	–	–	–	–	–	–
Interventions									
Active rTMS group	–	✓	✓	–	–	–	–	–	–
Sham rTMS group	–	✓	✓	–	–	–	–	–	–
Outcome measurement									
Postoperative resting pain score (NRS)	–	–	–	✓	✓	✓	✓	✓	✓
Postoperative movement pain scores (NRS)	–	–	–	✓	✓	✓	✓	✓	✓
Postoperative recovery quality index (QoR-15)	–	–	–	–	–	–	✓	✓	–
Remedial analgesic dosage (MME) in the first 48 h postoperatively	–	–	–	–	–	–	–	✓	–
Incidence of moderate to severe pain at 24 h postoperatively	–	–	–	–	–	–	✓	–	–
Adverse events	–	–	–	✓	✓	✓	✓	✓	✓

According to SPIRIT 2013 statement: defining standard protocol items for clinical trials. Pre-2, 2 days prior to the surgery; pre-1, the day before the surgery; MME, morphine mg equivalent; QoR-15, Quality of Recovery-15.

### 2.10 Outcomes

#### 2.10.1 Primary outcome

The primary outcome is the incidence of moderate-to-severe pain at 24 h postoperatively. APSP is common after VATS, often peaking within 24–48 h (32). Pain can inhibit breathing leading to atelectasis and impair postoperative mobility and physiotherapy (33). Pain intensity will be recorded using the Numerical Rating Scale (NRS) (34). An NRS score ≥ 4 is defined as moderate-to-severe pain (35). The primary outcome measure will be the NRS score for the most severe pain experienced within 24 h, recorded prior to any rescue analgesic administration.

#### 2.10.2 Secondary outcomes

The postoperative period is defined as starting from the time of PACU admission.

(1). Time to first PCIA demand (from surgery end to first effective PCA button press with drug delivery).
(2). Resting pain scores (NRS) at 2 h (T1), 6 h (T2), 12 h (T3), 24 h (T4), 48 h (T5), and 90 days (T6) postoperatively. Resting pain is assessed while the patient is lying quietly.
(3). Movement pain scores (NRS) at T1–T6 postoperatively. Movement pain is assessed during turning or coughing.
(4). PACU length of stay (hours from PACU admission to discharge).
(5). Area under the curve (AUC) for resting pain scores from T1 to T5 (0–48 h), calculated using the trapezoidal rule, to quantify total pain burden.
(6). AUC for movement pain scores from T1 to T5 (0–48 h), calculated using the trapezoidal rule.
(7). Quality of Recovery assessed by QoR-15 score at 24 h (T4) and 48 h (T5) postoperatively.
(8). Extubation time (minutes from cessation of all maintenance anesthetics to safe removal of the endotracheal tube). The decision to extubate, made by a blinded anesthesiologist, will be based on standard clinical criteria.
(9). Record the dosage of rescue analgesics administered within 48 h postoperatively, expressed in milligram equivalents of oral morphine (MME). Rescue analgesia is administered according to the protocol if the patient’s resting pain numerical rating scale (NRS) score is ≥4 within 48 h postoperatively. For standardized comparison, all opioid medications are converted to milligram equivalents of oral morphine based on the American Academy of Pain Medicine guidelines (36).

### 2.11 Adverse events and safety

All adverse events (AEs), regardless of relationship to rTMS, will be documented in detail, including time of onset, severity, relationship to intervention, actions taken, and outcome. SAEs must be reported to the Ethics Committee and principal investigator within 24 h, and participation in the study will be terminated. The study has a low risk of bias. An independent Data Monitoring Committee (DMC) will oversee the data, especially if adverse events occur. To address potential futility, the DMC will periodically review safety and efficacy data and perform an interim analysis when about 50% of data is collected. The O’Brien-Fleming alpha allocation function was employed, with the interim significance threshold set at *p* < 0.003. The interim analysis was conducted solely for safety and futility assessments and did not involve early termination for efficacy. If a negative reaction is suspected during the intervention, the study will be stopped immediately, and unblinding will occur quickly to ensure participant safety.

### 2.12 Data collection and management

Data will be recorded in Case Report Forms (CRFs) and include preoperative details like demographics, pulmonary function (FEV1/FVC), surgical type (segmentectomy, lobectomy, wedge resection, other), surgery duration, and intubation type (double-lumen tube or bronchial blocker). Postoperative data will include pulmonary function, NRS scores, QoR-15, delirium, incidence of postoperative nausea and vomiting, and gastrointestinal recovery. Data were collected and managed using REDCap, a secure electronic data capture system compliant with ICH-GCP and applicable regulatory requirements. CRFs were designed according to the study protocol and CDASH standards. Real-time data validation, including range checks and logical consistency, was programmed into the system. Discrepancies and queries were resolved by investigators through the EDC platform, with all modifications tracked by an audit trail. External laboratory data were imported electronically to reduce manual entry errors. Following completion of data collection, the database underwent a final review, was locked, and then exported for statistical analysis.

### 2.13 Sample size calculation

Our preliminary findings indicate a 70% incidence of moderate-to-severe pain postoperatively. Given prior studies reporting analgesic effects of rTMS over the M1 region, we hypothesize that a reduction in moderate-to-severe pain incidence to 50% following two interventions would constitute an effective therapeutic response (37). With α = 0.05 and β = 0.2 (80% power), the required sample size is 103 per group. Considering a 10% dropout rate and future subgroup analyses, we aim to recruit 260 patients, randomly assigned to active and sham stimulation groups.

### 2.14 Statistical analysis

Analysis will follow the intention-to-treat (ITT) principle using SPSS 27.0, with graphs created in GraphPad Prism 8.0. The Kolmogorov-Smirnov test will check normality, and Levene’s test will evaluate variance homogeneity. Data will be presented as mean ± standard deviation for normal distributions and median with interquartile range for non-normal distributions.

Continuous variables will be assessed with the *t*-test or Mann-Whitney U test, depending on distribution. Categorical variables, including the primary outcome, will be examined using chi-square tests or Fisher’s exact test, with differences shown as relative risk and 95% CI. Comparisons of primary outcomes used generalized estimating equations (GEE). The interaction between treatment and time was first assessed. If this interaction was statistically significant, Bonferroni-corrected multiple comparisons were performed to assess differences between groups at each individual time point. If not, the primary treatment effect was then assessed at each time point without applying the Bonferroni correction. For other replicated measurement data, GEE were similarly employed for analysis. Multivariate analysis will use linear or logistic regression models based on the outcome type. Missing data will be addressed with multiple imputation, and a per-protocol analysis will be included for sensitivity. Benjamini-Hochberg correction for secondary outcomes. A significance level of α = 0.05 will be applied, with *p* < 0.05 deemed significant.

## 3 Discussion

Video-assisted thoracoscopic surgery remains a primary therapeutic approach in thoracic surgery. Compared to open procedures, it offers significant advantages, including smaller incisions, reduced morbidity, accelerated recovery, diminished postoperative pain, and shorter hospital stays (38). However, VATS can lead to severe APSP that may develop into chronic pain lasting over 3 months (39). This type of pain is typically neuropathic and may manifest as referred pain in other parts of the body (40, 41). Postoperative pain following VATS represents a multifactorial and complex condition. It extends beyond minor incisional discomfort and results from the combined effects of chest tube irritation, nerve injury, manipulation of deep tissues, and musculoskeletal retraction (42). Furthermore, perioperative exposure to high-dose opioids may induce acute opioid tolerance and potentially opioid-induced hyperalgesia, while also altering the balance of descending inhibitory and facilitatory pathways originating from higher brain centers (43). Consequently, postoperative analgesia for VATS has emerged as a critical focus in the fields of anesthesiology and clinical surgery. Currently, the commonly employed postoperative analgesic modalities primarily include regional nerve blockade techniques, regional analgesic infusion techniques, and pharmacologic adjuncts. In regional nerve block techniques, the vertical erector spinae plane block is preferred over classic paravertebral and intercostal blocks because it is safer and less likely to harm critical structures. This technique is particularly suitable for patients undergoing anticoagulant therapy (44). New techniques such as the posterior superior serratus plane block are also undergoing clinical trials to validate their efficacy, offering physicians additional treatment options (45). For regional analgesia techniques involving indwelling catheters, the programmed intermittent epidural bolus (PIEB) modality demonstrates distinct advantages over traditional continuous infusion. By delivering anesthetic in a pulsed manner, PIEB facilitates more effective diffusion of the local anesthetic, resulting in a broader sensory block range and, to some extent, a reduction in overall drug consumption (46). Liposomal bupivacaine, a long-acting local anesthetic, provides sustained and more stable analgesia for up to 72 h when used for intercostal nerve blockade, effectively reducing patient pain scores. Adding intravenous dexamethasone and dexmedetomidine to nerve blocks reduces postoperative pain, opioid use, nausea, and vomiting, while improving recovery quality within 24–48 h (47). Virtual reality (VR) technology, as an innovative adjunct analgesic method, effectively distracts patients from pain and modulates pain perception by immersing them in virtual environments combined with cognitive behavioral therapy and breathing exercises. Studies indicate that combined VR analgesia significantly improves NRS scores within 48 h post-surgery (48). No single analgesic method is flawless, so multimodal analgesia is considered the gold standard.

Transcranial magnetic stimulation (TMS) is a safe, non-invasive technique that uses a changing magnetic field to create electrical currents in targeted brain areas, like the primary motor cortex, to alter neuron excitability. Due to its transient effects, a single session of TMS lacks clinical utility. rTMS involves the application of repetitive and rhythmic pulse stimuli to a specific brain region over an extended period, utilizing a fixed frequency and pattern (49). Currently, rTMS has become a relatively mature therapeutic approach in the treatment of neurological disorders and psychiatric conditions (50, 51). rTMS can cause long-term potentiation or depression, helping to reshape brain networks and showing promise in treating chronic pain. A 2015 meta-analysis found that high-frequency rTMS effectively reduces neuropathic pain, with multiple sessions enhancing its therapeutic impact (52). Research suggests that rTMS is a promising alternative to traditional drug therapy for managing pain in patients with various non-CNS cancers, including breast, non-small cell lung, gynecological cancers, multiple myeloma, and cellular glioma (53). A meta-analysis of 1,158 patients found that rTMS significantly reduced depression scores and enhanced follow-up outcomes and quality of life in those with musculoskeletal pain (54). Zhong et al. demonstrated that neural modulation through rTMS is significantly correlated with the alleviation of chronic phantom limb pain (55). Further research indicates that stimulating the dorsolateral prefrontal cortex (DLPFC) can also produce a measurable analgesic effect (56). The DLPFC is a crucial component of the prefrontal cortex. Rather than directly processing the physical signals of pain, it functions as the brain’s higher-order command center, deeply involved in cognitive control, emotional regulation, and attention allocation. By regulating the emotional and affective dimensions of pain, it indirectly suppresses excessive negative emotional responses in downstream limbic systems, thereby alleviating the emotional distress associated with pain (57). Studies have demonstrated that stimulating the DLPFC with rTMS can prevent migraine attacks (58). The findings of Che et al. provide evidence that high-frequency stimulation of the DLPFC can elicit analgesic effects in both chronic pain conditions and in response to experimentally induced pain (59). In summary, rTMS demonstrates considerable potential in alleviating chronic pain.

The feasibility of rTMS in alleviating APSP has recently emerged as a research focus. The theoretical basis for this lies in the fact that the conscious perception of pain is ultimately integrated by the cerebral cortex. High-frequency stimulation of key pain matrix nodes, like the M1 area, through rTMS can activate natural pain relief pathways and alter brain activity in regions like the anterior cingulate cortex and insula, disrupting acute pain signal processing. rTMS enhances the excitability of cortical neurons over the long term, strengthens the cortical structures involved in descending pain inhibition, and suppresses central sensitization, thereby reducing patients’ perception of pain. This provides a theoretical basis for the preventive use of rTMS to alleviate postoperative pain (60). Animal experiments have shown that rTMS can increase the levels of brain-derived neurotrophic factor in the prefrontal cortex, which helps promote neuroplasticity and allows the brain to adapt and respond to upcoming pain stimuli in a healthier manner (61). Compared to traditional pharmacologic analgesic approaches, rTMS offers unique advantages including the absence of systemic side effects, no drug-drug interactions, and high patient acceptance. Although this field is still in its exploratory stages and requires more large-scale randomized controlled trials to optimize stimulation protocols and confirm the durability of its efficacy, preliminary clinical evidence suggests that administering a single session or a short course of rTMS treatment to patients, either before or after surgery, holds promise as an effective adjunct analgesic modality. This approach provides a new non-drug option for creating multimodal postoperative pain relief plans (62–64).

The NRS is a widely used pain assessment tool with a 0–10 scale, where 0 indicates no pain and 10 indicates the worst possible pain (65). In this study, pain with an NRS score of 4 or higher was considered moderate to severe. Reports indicate that 50%–70% of patients experience such pain within 24–48 h after surgery. Thus, the occurrence of moderate-to-severe pain at 24 h post-surgery is a suitable primary outcome measure for this study (66). As no previous studies have explored rTMS’s impact on APSP, we based our stimulation parameters on preclinical findings from standard rTMS research (67). A meta-analysis of rTMS and pain-related research indicated that 12 out of 15 studies targeted the primary motor cortex (68). In clinical studies, high-frequency stimulation (10 Hz) rTMS is used for pain treatment, typically at 80%–90% RMT (69, 70). For safety and efficacy, we chose a 10 Hz, 3,000-pulse stimulation protocol for the M1 region (71).

There are several limitations to note: the study’s single-center nature and modest sample size may limit generalizability; the limited range of VATS procedures at our institution might restrict broader applicability; fixed stimulation parameters were used without exploring dose-response relationships; and objective biomarkers for postoperative pain recovery were not included.

While preclinical research on rTMS for perioperative pain relief is developing, clinical evidence is still scarce. This study examines the effects of this non-invasive technique on APSP in VATS patients, offering a new angle on multimodal pain management and potentially enhancing postoperative recovery.
