# Supplementary material for: Efficacy of repetitive transcranial magnetic stimulation on postoperative pain in patients undergoing video-assisted thoracoscopic surgery: study protocol for a prospective, single-center, randomized controlled trial
Source: Front Med (Lausanne). 2026 Mar 19;13:1756881. doi: 10.3389/fmed.2026.1756881 (PMC13044022; doi:10.3389/fmed.2026.1756881)
Supplement: Supplementary file 1 [file Table_1.docx]

The Effect of Repetitive Transcranial Magnetic Stimulation on Postoperative Pain in Patients Undergoing Thoracoscopic Surgery: A Prospective, Double-Blind, Randomized Controlled Trial

**Informed Consent Form**

Dear Participant,

We invite you to participate in the study titled “The Effect of Repetitive Transcranial Magnetic Stimulation on Postoperative Pain in Thoracoscopic Surgery Patients: A Prospective, Double-Blind, Randomized Controlled Trial,” approved by the Medical Ethics Committee of Jiaxing First Hospital. This study will be conducted at Jiaxing First Hospital, with an estimated 260 participants expected to voluntarily enroll.

Portions of this document are required by law and have been reviewed and approved by the Medical Ethics Committee to protect the rights and interests of participating patients.

**1. Why conduct this study?**

Research Background: The high incidence of moderate-to-severe acute pain following video-assisted thoracoscopic surgery (VATS) significantly impacts patient recovery and quality of life. Despite widespread adoption of multimodal analgesic strategies, their side effects and limitations remain noteworthy. Repetitive transcranial magnetic stimulation (rTMS) is a non-invasive neuromodulation technique. High-frequency stimulation of the primary motor cortex (M1 region) has been demonstrated to activate endogenous descending pain inhibitory pathways, providing effective analgesia in chronic pain management. However, its application in preventing and treating acute postoperative pain, particularly following VATS, requires high-level evidence-based medical support.

Research Objectives:

(1) To investigate the impact of preoperative rTMS on postoperative pain: Explore the association between preoperative rTMS use and the incidence of moderate-to-severe postoperative pain, and attempt to determine the magnitude of rTMS stimulation's effect on postoperative pain.

(2) To examine the current status of postoperative pain occurrence in VATS patients and analyze its influencing factors.

Study Scope:

All patients participating in this study were from the Department of Cardiothoracic Surgery at our hospital.

**2. How is this study conducted?**

We will obtain information about your health or medical conditions through electronic medical records and laboratory tests. Throughout the study, we will collect and inform you of your health status through a series of examinations and procedures.

**3. What will I need to do during the study?**

This study has been reviewed and approved by the Medical Ethics Committee of Jiaxing First Hospital. Beyond your routine medical care and examinations at our hospital, please inform us of any changes during your visits. We will collect relevant test results and changes in your condition, such as blood biochemistry indicators, without requiring additional tests or examinations.

**4. How will participating in this study affect my life?**

You may find these examinations inconvenient and require special arrangements. Additionally, some procedures, such as rTMS treatment, may cause discomfort. If you have any questions about the examinations or steps involved in the study, please consult the study physician.

**5. What risks and adverse reactions might I experience by participating in this study?**

You may experience adverse reactions during the study. We will monitor all patients for any adverse reactions. If you experience any adverse reactions, please consult your study doctor promptly.

Currently, medications used during routine clinical care in this study may cause the following discomforts:

1. Dizziness caused by anesthetic drugs.

2. Nausea and vomiting caused by postoperative analgesic drugs. Discomfort associated with routine diagnostic tests may include: 1. Discomfort from pulmonary function tests. 2. Pain at the stimulation site from rTMS treatment, etc.

You should inform your family or close friends that you are participating in a prospective, randomized, double-blind, stratified study. They can be aware of the events described above. If they have questions about your participation, you can tell them how to contact your study doctor.

**6. What benefits might I gain from this study?**

Participation in this study may lead to improved postoperative pain management.

**7. What compensation will I receive for participating in this study?**

You will not receive any monetary compensation for participating in this study.

**8. What happens if I am harmed during the study? How can harm be prevented, and what treatment measures are available if harm occurs?**

If you experience any research-related harm to your health due to participation in this study, please notify the study physician immediately. They will be responsible for providing appropriate treatment measures in accordance with national regulations. For injuries resulting from non-compliance with study protocols or from your own poor diet or lifestyle choices, any medical disputes will be handled according to relevant laws and regulations.

Even after signing this informed consent form, you retain all your legal rights.

**9. Is my personal information confidential?**

Your medical records will be kept at the hospital. Researchers, the research administration department, and the ethics committee will be permitted to access your medical records. Any public reports regarding the results of this study will not disclose your personal identity. We will make every effort to protect the privacy of your personal medical information within the scope permitted by law.

By signing this informed consent form, you agree that your personal and medical information may be used for the purposes described above.

**10. Do I have to participate in the study? Are there alternatives?**

Participation in this study is entirely voluntary. You may refuse to participate or withdraw from the study at any time during its course without providing any reason. This decision will not affect your standard treatment.

If you decide to withdraw from the study, please notify your study physician in advance. For your safety, you may be asked to undergo certain examinations, which are beneficial for protecting your health.

There are no alternative options to this study.

**Subject Consent Statement:**

I have read the above information regarding this study and fully understand the potential risks and benefits of participating. I voluntarily consent to participate in the clinical research described herein.

I consent □ do not consent □ to the use of my medical records and pathology specimens in studies other than this one.

Subject Signature: Date:

Printed Name: Phone Number:

(Note: If the subject is legally incapacitated or lacks capacity, a legal guardian must sign.)

Legal Guardian Signature: Signature Date:

Legal Guardian Name (Print): Legal Guardian Contact Number:

(Note: If the subject is unable to read, a fair witness must sign.)

Fair Witness Signature: Signature Date:

Fair Witness Name (Print): Fair Witness Contact Number:

**Researcher Declaration:** I confirm that I have explained the details of this study to the patient, particularly the potential risks and benefits of participating.

Researcher Signature: Date:

Researcher Name (Printed): Contact Number:

Contact Information for the Medical Ethics Committee of Jiaxing First Hospital:

Jiaxing First Hospital, 1882 Zhonghuan South Road, Jiaxing City, Zhejiang Province

Tel: 0573-89976378
